# Correction: Multivariate analysis of electrophysiological diversity of *Xenopus* visual neurons during development and plasticity

**DOI:** 10.7554/eLife.14282

**Published:** 2016-01-28

**Authors:** Christopher M Ciarleglio, Arseny S Khakhalin, Angelia F Wang, Alexander C Constantino, Sarah P Yip, Carlos D Aizenman

Christopher M Ciarleglio, Arseny S Khakhalin, Angelia F Wang, Alexander C Constantino, Sarah P Yip, Carlos D Aizenman, Ciarleglio CM, Khakhalin AS, Wang AF, Constantino AC, Yip SP, Aizenman CD. 2015. Multivariate analysis of electrophysiological diversity of *Xenopus* visual neurons during development and plasticity. *eLife* **4**:e11351. doi: 10.7554/eLife.11351.

Published 14 November 2015

In the published article, all authors were indicated as being affiliated with both Bard College and Brown University. However only Arseny Khakhalin should have been affiliated with both these affiliations. The other authors should have been affiliated with Brown University only.

The article has been corrected accordingly.

