# Supplementary material for: Multivariate analysis of electrophysiological diversity of Xenopus visual neurons during development and plasticity
Source: eLife. 2015 Nov 14;4:e11351. doi: 10.7554/eLife.11351 (PMC4728129; doi:10.7554/eLife.11351)
Supplement: Supplementary file 1. — 33 variables were extracted from this dataset, the table describes the name of the variables, units. average value and brief description. For more details about each variable please see Materials and methods. DOI: http://dx.doi.org/10.7554/eLife.11351.011 [file elife-11351-supp1.docx]

**Supplementary File 1: Table of variables**

| **#** | **Short name** | **Units** | **Average value in naïve set** | **N** | **Brief description** (See "Methods" for a detailed description) |
| --- | --- | --- | --- | --- | --- |
| 1 | Cm | pF | 14.8 ± 3.6 | 154 | Cell membrane capacitance |
| 2 | Rm | GΩ | 1.2 ± 0.6 | 153 | Cell membrane resistance |
| 3 | Ra | MΩ | 53.9 ± 23.1 | 153 | Access resistance |
| 4 | I hold | pA | 15.8 ± 13.4 | 151 | Current required to voltage-clamp the cell at −65 mV |
| 5 | Na activation | mV | −20.3 ± 6.2 | 154 | Lowest potential that triggered voltage-gated Na current |
| 6 | I_Na_ | pA | 434 ± 226 | 154 | Maximal voltage-gated Na current |
| 7 | KS activation | mV | −19.0 ± 6.4 | 154 | Lowest potential to trigger slow voltage-gated K current |
| 8 | I_KS_ | pA | 537 ± 259 | 154 | Maximal voltage-gated slow K current |
| 9 | KT activation | mV | −15.6 ±11.7 | 154 | Lowest potential to trigger transient voltage-gated K current |
| 10 | I_KT_ | pA | 171 ± 98 | 154 | Maximal voltage-gated transient K current |
| 11 | Tail | ms | 41 ± 16 | 135 | Time constant of cell repolarization after step injection (the prominence of a tail potential after step injection) |
| 12 | Spike threshold | mV | −25.0 ± 7.6 | 134 | The potential of a "kink point", at which neuron switched from passive to "explosive" response to current injection. |
| 13 | Spike amplitude | mV | 19.9 ± 10.2 | 134 | Spike amplitude in current clamp mode |
| 14 | Spike rise-time | ms | 1.5 ± 1.0 | 134 | Spike rise time in current clamp mode |
| 15 | Spike width | ms | 4.0 ± 2.4 | 134 | Spike width |
| 16 | I best | pA | 122 ± 52 | 134 | Steady current injection that produced highest spiking |
| 17 | N spikes, step | n | 4.5 ± 3.1 | 134 | Max. number of spikes produced on step injection |
| 18 | Spike ISI | ms | 13.9 ± 6.0 | 117 | Inter-spike interval for best step injection |
| 19 | Spike ISI accomm | - | 1.1 ± 0.2 | 90 | Ratio of 2nd and 2st inter-spike intervals |
| 20 | Spike accomm. | - | 2.3 ± 1.3 | 117 | Spike amplitude accommodation. |
| 21 | N spikes, cosine | n | 0.7 ± 0.4 | 108 | Max. number of spikes produced on cosine injection |
| 22 | Spiking resonance | ms | 43 ± 10 | 108 | Cosine injection period that produced highest spiking |
| 23 | Spiking resonance width | pA | 53 ± 52 | 108 | A measure of non-saturation of spike output in response to slower cosine injections (a measure of non-inactivation) |
| 24 | Wave buildup | n | 9.7 ± 5.8 | 108 | Cosine "bump" by which the cell reached highest spiking |
| 25 | Wave decay | n | 35 ± 64 | 108 | A measure of spiking inactivation: decay constant for spiking in response to consecutive cosine "bumps" |
| 26 | Jitter | - | 0.1 ± 0.1 | 108 | Spike-timing jitter coefficient during cosine injections |
| 27 | Synaptic resonance | ms | 52.5 ± 76.3 | 76 | Inter-stimulus interval that evoked maximal total synaptic response (total charge) |
| 28 | Synaptic resonance width | ms | 90 ± 85 | 76 | Sharpness of non-linear summation as a function of inter-stimulus interval (high value = low dependency) |
| 29 | Synaptic charge | nA·s | 16.8 ± 18.0 | 76 | Maximal total synaptic charge |
| 30 | Synaptic PPF | - | 2.4 ± 1.5 | 76 | A measure of synaptic non-linear summation |
| 31 | Monosynapticity | - | 3.1 ± 2.8 | 76 | Ratio of monosynaptic to polysynaptic responses |
| 32 | Minis frequency | Hz | 4.8 ± 4.4 | 64 | Frequency of spontaneous excitatory postsynaptic currents |
| 33 | Minis amplitude | pA | 5.0 ± 2.1 | 64 | Mean amplitude of excitatory postsynaptic currents |
