# Supplementary material for: Multivariate analysis of electrophysiological diversity of Xenopus visual neurons during development and plasticity
Source: eLife. 2015 Nov 14;4:e11351. doi: 10.7554/eLife.11351 (PMC4728129; doi:10.7554/eLife.11351)
Supplement: Supplementary file 3. — The data in this table describes the average values of each measured variable across several cells grouped by developmental stage and experimental condition. DOI: http://dx.doi.org/10.7554/eLife.11351.013 [file elife-11351-supp3.docx]

## Supplementary File 3. Average values for different stages and experimental conditions

|  |  | | **naïve** | | | | | | **Stimulated** | |
| --- | --- | --- | --- | --- | --- | --- | --- | --- | --- | --- |
| **N** | **Name, units** | | **Stage 43-44** | **Stage 45-46** | **Stage 47** | **Stage 48-49** | **pd** | **pv** | **Stage 48-49** | **ps** |
| 1 | Cm | pF | 14.0 ± 1.9 | 15.7 ± 3.1 | *15.7 ± 2.7* | 13.6 ± 4.5 | .. | .. | 11.6 ± 4.9 | .. |
| 2 | Rm | GΩ | 1.0 ± 0.2 | 1.2 ± 0.6 | 1.2 ± 0.4 | 1.3 ± 0.7 |  |  | 1.3 ± 1.1 |  |
| 3 | Ra | MΩ | 45.7 ± 15.2 | 54.0 ± 15.8 | 57.9 ± 24.1 | 53.7 ± 30.2 |  | ** | 44.0 ± 23.7 |  |
| 4 | I hold | pA | 20.3 ± 12.9 | 14.9 ± 10.5 | 11.1 ± 12.1 | 17.9 ± 16.5 |  | ** | 29.4 ± 20.2 | * |
| 5 | Na activation | mV | −22.6 ± 5.6 | −22.1 ± 6.4 | −17.3 ± 4.0 | −19.0 ± 6.0 | * |  | −18.8 ± 7.2 |  |
| 6 | I_Na_ | pA | 403 ± 160 | 458 ± 165 | 488 ± 228 | 386 ± 284 |  | ** | 351 ± 168 |  |
| 7 | KS activation | mV | −21.8 ± 7.7 | −19.0 ± 5.9 | −16.4 ± 5.8 | −19.4 ± 6.7 |  |  | −18.5 ± 8.4 |  |
| 8 | I_KS_ | pA | 535 ± 203 | 494 ± 182 | 502 ± 201 | 603 ± 344 |  | ** | 539 ± 263 |  |
| 9 | KT activation | mV | −23.6 ± 3.8 | −18.0 ± 10.0 | −15.2 ± 4.2 | −11.5 ± 15.0 | * | * | −14.8 ± 9.0 |  |
| 10 | I_KT_ | pA | 172 ± 60 | 167 ± 90 | 241 ± 127 | 144 ± 88 | ** |  | 147 ± 80 |  |
| 11 | Tail | ms | 44.1 ± 11.3 | 38.4 ± 12.8 | 40.1 ± 15.4 | 42.1 ± 20.6 |  | * | 43.7 ± 18.6 |  |
| 12 | Spike threshold | mV | −18.7 ± 6.4 | −25.8 ± 7.1 | −25.9 ± 6.7 | −25.2 ± 8.1 | . |  | −19.8 ± 5.3 | ** |
| 13 | Spike amplitude | mV | 14.5 ± 6.8 | 20.0 ± 8.8 | 21.4 ± 10.1 | 20.3 ± 12.0 |  | . | 28.3 ± 14.0 | * |
| 14 | Spike rise-time | ms | 2.4 ± 1.8 | 1.5 ± 0.9 | 1.1 ± 0.5 | 1.5 ± 0.9 | * |  | 1.3 ± 0.8 |  |
| 15 | Spike width | ms | 5.4 ± 2.1 | 4.2 ± 2.3 | 2.7 ± 1.9 | 3.9 ± 2.5 | . |  | 3.4 ± 2.4 |  |
| 16 | I best | pA | 100.6 ± 53.4 | 112.7 ± 51.6 | 146.9 ± 42.9 | 125.2 ± 52.3 | . |  | 108.8 ± 49.4 |  |
| 17 | N spikes, step | n | 2.3 ± 0.8 | 3.9 ± 2.6 | 6.7 ± 3.6 | 4.6 ± 3.3 | ** |  | 7.1 ± 4.7 | .. |
| 18 | Spike ISI | ms | 16.0 ± 6.6 | 14.9 ± 6.4 | 12.2 ± 5.4 | 13.1 ± 5.6 |  |  | 13.9 ± 6.7 |  |
| 19 | Spike ISI accomm | - | 1.1 ± 0.3 | 1.1 ± 0.3 | 1.0 ± 0.2 | 1.1 ± 0.2 |  |  | 1.1 ± 0.3 |  |
| 20 | Spike accomm. | - | 2.2 ± 0.8 | 2.4 ± 1.3 | 1.8 ± 0.9 | 2.5 ± 1.5 |  |  | 2.4 ± 1.2 |  |
| 21 | N spikes, cosine | n | 0.3 ± 0.2 | 0.7 ± 0.3 | 1.1 ± 0.3 | 0.8 ± 0.5 | ** | . | 0.9 ± 0.5 |  |
| 22 | Spiking resonance | ms | 47.7 ± 5.6 | 43.9 ± 8.1 | 40.2 ± 11.9 | 40.9 ± 11.3 |  | . | 42.7 ± 10.8 |  |
| 23 | Spiking resonance width | pA | 50.3 ± 36.4 | 41.8 ± 44.8 | 62.8 ± 62.9 | 61.2 ± 56.6 |  |  | 77.0 ± 56.8 |  |
| 24 | Wave buildup | n | 10.0 ± 5.6 | 9.2 ± 5.8 | 10.1 ± 6.8 | 10.1 ± 5.8 |  |  | 7.4 ± 5.3 | .. |
| 25 | Wave decay | n | 19.3 ± 60.0 | 8.3 ± 24.4 | 37.1 ± 55.1 | 67.6 ± 83.1 | ** | ** | 81.1 ± 89.2 |  |
| 26 | Jitter | - | 0.2 ± 0.1 | 0.1 ± 0.1 | 0.1 ± 0.0 | 0.1 ± 0.1 |  |  | 0.1 ± 0.1 | . |
| 27 | Synaptic resonance | ms | 23.1 ± 27.6 | 75.6 ± 89.8 | 52.3 ± 75.4 | 30.7 ± 58.4 |  | . | 20.4 ± 22.1 |  |
| 28 | Synaptic resonance width | ms | 47.2 ± 57.5 | 124.1 ± 100.6 | 75.0 ± 76.6 | 66.3 ± 59.8 | . | . | 81.3 ± 87.2 |  |
| 29 | Synaptic charge | nA·s | 11.2 ± 3.8 | 13.1 ± 11.7 | 20.5 ± 24.0 | 20.0 ± 21.0 |  | * | 26.3 ± 22.4 |  |
| 30 | Synaptic PPF | - | 2.2 ± 1.3 | 2.0 ± 1.2 | 2.2 ± 1.2 | 3.0 ± 1.9 |  | . | 2.6 ± 2.1 |  |
| 31 | Monosynapticity | - | 2.5 ± 1.3 | 3.6 ± 3.5 | 2.1 ± 1.2 | 3.2 ± 2.7 |  |  | 2.9 ± 3.7 |  |
| 32 | Minis frequency | Hz | 3.4 ± 2.3 | 4.1 ± 3.4 | 6.5 ± 3.8 | 5.1 ± 6.3 |  | .. | 3.2 ± 2.4 |  |
| 33 | Minis amplitude | pA | 4.1 ± 1.1 | 5.2 ± 1.4 | 5.5 ± 3.2 | 4.6 ± 1.9 |  |  | 4.1 ± 1.3 |  |

Key:

**pd**: *P*-value for changes in cell parameters over development, calculated as ANOVA test over developmental stages grouped as shown in the table (stages 43-44, 45-46, 47, and 48-49); not corrected for multiple comparisons.

**pv**: *P*-value for changes in cell variability between stages 45-46 and 48-49 (F-test for the inequality of variances).

**ps**: *P*-value for changes between naïve and stimulated cells from stage 48-49 tadpoles (*P*_MW_).

*P*-values key: ** < 0.001, * < 0.005, .. < 0.01, . < 0.05. Not adjusted for multiple comparisons.
