# Supplementary material for: Multivariate analysis of electrophysiological diversity of Xenopus visual neurons during development and plasticity
Source: eLife. 2015 Nov 14;4:e11351. doi: 10.7554/eLife.11351 (PMC4728129; doi:10.7554/eLife.11351)
Supplement: Source code 1. — This set of custom functions was not designed for public use, so many aspects of data analysis in these functions are hard-coded (usually as a global variable explicitly defined in the beginning of each script). Note also that at the submission stage we transformed all raw data for this paper from pClamp ABF files to Matlab MAT-file format, and this is how the data was uploaded to Dryad. The original scripts however worked with our original data files that were mostly stored in either ABF or Microsoft Excel formats. DOI: http://dx.doi.org/10.7554/eLife.11351.014 [file elife-11351-code1.zip › shared code/Code readme (Ciarleglio Khakhalin Aizenman).docx]

Source Code to accompany
(Ciarleglio, Khakhalin, Aizenman)

# Description

This file contains a description of Matlab and R scripts that were used to analyze data for the publication "Multivariate analysis of electrophysiological diversity of Xenopus visual neurons during development and plasticity" by C.M. Ciarleglio, A.S. Khakhalin, C.D. Aizenman et. al. This set of custom functions was not designed for public use, so many aspects of data analysis in these functions are hard-coded (usually as a global variable explicitly defined in the beginning of each script). Note also that at the submission stage we transformed all raw data for this paper from pClamp ABF files to Matlab MAT-file format, and this is how the data was uploaded to Dryad. The original scripts however worked with our original data files that were mostly stored in either ABF or Microsoft Excel formats.

All questions about these scripts should be addressed to A.S. Khakhalin ([khakhalin@bard.edu](mailto:khakhalin@bard.edu)).

## Matlab scripts

| **Script name** | **Description** |
| --- | --- |
| chris_NaK.m | This script processes and visualizes raw data for IV curves, and fits them with appropriate fit functions. |
| chris_injection.m | Analyzes and visualizes spike-timing data for step current injections. Fits each set of data with a curve and returns estimations of spikiness. |
| chris_detect_spikes.m | Spike detection script. It reads raw current clamp files with current injections one by one, detects spikes in each of them, and either saves them as a TXT files, or outputs spike times into console. Can also output several types of plots that are used for visual debugging of the algorithm. |
| chris_step_inject_juggler.m | Extracts spike-timing parameters (such as inter-spike interval and inter-spike accommodation) from the spike-timing data. |
| chris_step_manual_gui.m | A program to manually browse step injection data and check whether the spikes were identified properly. |
| chris_step_inject_browser.m | An interactive tool that reads all raw data for step current injection, links them to a PCA scoreplot, and allows the user to browse through the raw data by clicking at respective markers on the scoreplot. |
| chris_step_inject_browser2.m | Builds a PCA-like plot (scoreplot) with spiking responses to current step injection instead of markers. PCA results and the list of cells are hard-coded in the text of the function. |
| chris_osc_jitter.m | Calculates jitter for spiking responses to cosine current injections. |
| chris_osc_fit.m | Summarizes and fits information about spiking in response to cosine current injections; calculates a set of high-order spiking parameters for each cell. |
| chris_pca.m | Runs standard (classic) PCA on a subset of data with all values available. |
| chris_pca6.m | The main PCA-with-missing-values file. Runs as a script, not a function, leaving all important reusable information in the workspace. Relies on the external library "pca_full" by Alexander Ilin and Tapani Raiko (see the main text for details). Contains multiple sections that can be triggered ON and OFF, and that consecutively process different aspects of the data, including: main PCA analysis; PCA on rank-transformed data; comparison of naïve and stimulated cells; projection of stimulated cells into naïve PCA space; PCA on a subset of variables defined by principal variables analysis; different types of post-PCA rotation; generation of loading plots and several types of scoreplots; analysis of variables across developmental stages; analysis of variables between naïve and stimulated cells; comparison of variability of data in the original 33D space; comparison of variance explained (eta squared effect size) across development and after stimulation; correlation analysis (including a circos-like plot, which relies on the circos.m function, and FDR for correlations); sensitivity analysis of cell location, time of the experiment, and preparation age. |
| circos.m | Circos-inspired circular visualization of correlations between individual variables. |
| chris_color_age_pca4 | Builds the figure that illustrates the evolution of the PCA results across developmental stages. Relies on an external library "kde2d" (see the main text for details). |
| chris_bursts2.m | Processes synaptic burst data; outputs quantification variables for each cell. |
| chris_burst_manual_gui.m | A program to manually browse synaptic bursts. |
| chris_false_twins2.m | Loads spiking data, finds pairs of similar cells, and for each "seed cell" finds several sells with spiking "most similar" to that of a "seed cell". Relies on the code by Victor & Purpura 1996, which in this case is included as a subfunction within the same file. |
| chris_color_age_pca5.m | Shows how pre-set groups of cells with similar spiking patterns (from the "false twins" program) project into the PCA scoreplot. Many aspects of the data are hard-coded in the program text. |

## Auxiliary Matlab scripts

To increase the possibility of code reuse, we also include several technical functions that were used for creation of these scripts. As these functions are not critical for the understanding of how the scripts work, they are included without comments.

The list of auxiliary functions included: enum, circle, compare_columns, values_and_averages3, supertitle, ribbonplot, concatenan, dispf, fdr.

## R scripts

| principal_variables.R | Performs the principal variables analysis (package "subselect"). |
| --- | --- |
| imputation.R | Performs multiple imputation on the data (package "mi"), and then combines methods of agglomerative clustering (package "agnes"), cluster quantification (standard cluster analysis in R), multidimensional scaling (function "cmdscale"), isomapping (function "isomap"), and local linear embedding (package "lle"). |
